# Engineering Resilience: How Irradiation Strategies Influence 3D-Bioprinted Adipose Stem Cells

**DOI:** 10.3390/bioengineering13010025

**Published:** 2025-12-26

**Authors:** Nicki Amiri, Rafael Schmid, Stefan Schrüfer, Zan Lamberger, Philipp Stahlhut, Gregor Lang, Yvonne Kulicke, Andreas Arkudas, Raymund E. Horch, Wibke Müller-Seubert

**Affiliations:** 1 Laboratory for Tissue Engineering and Regenerative Medicine, Department of Plastic and Hand Surgery, University Hospital Erlangen, Friedrich Alexander University Erlangen-Nürnberg FAU, Krankenhausstr. 12, D-91054 Erlangen, Germany; 2 Institute of Polymer Materials, Friedrich-Alexander University Erlangen-Nürnberg, Martensstraße 7, D-91058 Erlangen, Germany; 3 Department for Functional Materials in Medicine and Dentistry, University Hospital of Würzburg, Pleicherwall 2, D-97070 Würzburg, Germany

**Keywords:** cell viability, irradiation, biofabrication, bioink, adipose-derived stem cells, mesenchymal stem cells, tissue engineering

## Abstract

Background: Reconstructive defect coverage after irradiation remains a challenge in reconstructive surgery, as ionizing radiation leads to tissue ischemia and fibrosis. Therefore, the application of adipose-derived stem cells (ASCs) might be a therapeutic strategy for improving flap survival. Nevertheless, the influence of irradiation on ASCs remains unclear. This study examines the effect of irradiation on 3D-printed ASCs. Methods: ASCs were 3D-cultured. The constructs were irradiated with 2 Gy and 5 Gy; one group treated with 0 Gy served as the non-irradiated control group. Cell viability was measured via a WST-8 assay, a live/dead assay and fluorescence microscopy 24 h, 48 h and 72 h after irradiation. Furthermore, qPCR analysis was performed to detect the expression of HIF-1α, p53 and IL-6 at the same timepoints. Results: Cell survival was high after 24 h. Expression of HIF1α after 24 h was 1.5 times significantly higher in the 2 Gy group compared with the 0 Gy group. The expression of other genes was not significantly affected by irradiation. Measurement of the metabolic activity and survival of the ASCs did not show differences between the different groups at all timepoints. Conclusions: 3D-cultured adipose-derived stem cells maintain high viability after moderate irradiation, suggesting radioresistance.

## 1. Introduction

Reconstructive defect coverage following oncological resection remains a fundamental component of plastic and reconstructive surgery. However, these complex surgical interventions are significantly more complicated when patients have undergone radiotherapy preoperatively or are scheduled to receive it postoperatively. Ionizing radiation induces microvascular injury, leading to tissue ischemia and subsequently promoting progressive fibrosis of the irradiated area [1]. Inflammation, endothelial cell injuries and fibrosis are the most significant complications of this procedure [2]. These pathophysiological alterations markedly impair the regenerative capacity of the tissue and hinder the successful integration of grafts or flaps. In this context, regenerative therapeutic strategies aimed at preserving or restoring cellular proliferative potential and enhancing local microcirculation are gaining increasing importance in both translational research and clinical applications.

The therapeutic application of adipose-derived stem cells (ASCs) has emerged as a central focus in regenerative strategies aimed at enhancing flap viability. ASCs are mesenchymal stem cells (MSCs) that are obtained from adipose tissue and are found abundantly in the human body. Therefore, they can be isolated in high numbers [3]. They were first identified as MSCs in adipose tissue in 2001, and since then, they have been studied as a cell source for tissue engineering and regenerative medicine [4]. Due to their multi-lineage capacity, they offer the potential to repair, maintain or enhance various tissues [3]. Recent studies have shown that the use of mesenchymal stem cells represents a relevant strategy in regenerative medicine due to their regenerative character and paracrine-secreting ability, which influences nearby organs positively [5,6]. ASCs have shown especially promising results in regenerating tissues and organs damaged by injury [7].

Nevertheless, the majority of existing studies have not accounted for the impact of ionizing radiation on stem-cell-based therapies. Irradiation has been reported to reduce the proliferation rate of ASCs [8,9,10]. However, these findings are predominantly derived from conventional two-dimensional (2D) cell culture systems, which fail to replicate the structural and functional complexity of native tissue environments. In contrast, three-dimensional (3D) cell culture platforms offer a more physiologically relevant model for studying key biological processes, including cell proliferation, metabolic activity and morphological characteristics [11]. Studies have shown that 3D cultivation of cells enables one to study the direct influence of different microenvironmental factors on the behavior of different cell types [11].

3D bioprinting enables the precise spatial organization of cells within biocompatible hydrogels, allowing the fabrication of constructs that replicate native tissue structures and cellular microenvironments. Several studies have highlighted the versatility of bioprinting and the potential of hydrogel-based printing to generate complex and physiologically relevant tissue models [12,13,14]. In addition, 3D bioprinting provides a high degree of reproducibility and efficient production, ensuring reliable experimental outcomes.

This precise fabrication process allows for accurate specifications and has the potential to greatly benefit cancer treatment and other research because it could offer medical solutions tailored to each individual patient. In particular, if the bioprinted constructs are engrafted with stem cells, they could serve as personalized implants for regenerative medicine and in vitro high-throughput drug development models for drug discovery [15,16].

Even though bioprinting is likely to revolutionize the field of medicine in the future, one of the most significant challenges in bioprinting is the integration of functionality into printed structures. Bioprinting seeks to replicate not only the form but also the dynamic functionality of biological systems. As natural organisms can adapt to their environment and perform complex functions such as self-healing or regeneration, bioprinting needs to integrate these dynamic adaptive behaviors into 3D-printed structures [17]. Bioprinting combines expertise from engineering, materials science and cell biology, and for this reason, unique challenges in determining the ownership and patentability of innovations might be relevant in the future [18].

Among the available biomaterials, gelatin methacryloyl (GelMA) has emerged as a promising bioink due to its tunable mechanical properties, printability and natural cell-binding motifs [12]. In previous studies, we have demonstrated that constructs made with gelatin-based hydrogels were better at maintaining cell morphology and proliferation compared with using other materials, supporting the motivation for choosing GelMA as a bioink in this study [19].

Before bringing stem cell therapy into daily clinical practice, the present study aims to systematically investigate the effects of ionizing radiation on three-dimensionally printed cell-laden constructs. The focus is on characterizing both functional and morphological alterations in irradiated cell populations within biofabricated architectures. Through this analysis, fundamental insights into the radiobiological responses of stem cells under 3D culture conditions are to be obtained, with particular emphasis on their capacity to support neovascularization in irradiated or radiation-planned tissue.

Ultimately, the results of this investigation are intended to inform the development of targeted regenerative strategies aimed at enhancing tissue repair and vascular integration in regions compromised by radiotherapy, thereby expanding the therapeutic potential of cell-based interventions in reconstructive surgery.

## 2. Materials and Methods

### 2.1. Cell Culture

For these experiments, immortalized adipose-derived stem cells (ASCs) named ASC52telo from Everycyte (Everycyte GmbH, Vienna, Austria) were used. These were cultivated in an endothelial cell basal medium-2 (EBM-2) by Clonetics, Lonza Group AG (Basel, Switzerland), which was enriched with the corresponding supplement mix without Gentamicin Sulfate Amphotericin-B (GA-1000), 2% fetal calf serum superior (FCS) by Sigma Aldrich (St. Louis, MO, USA) and 200 μg/mL geneticin from Sigma Aldrich. The cells were incubated at 37 °C along with 5% CO_2_, and the medium was changed every 2 days. To maintain optimal growth and to obtain the amount needed for printing, the cells were subcultured every week.

### 2.2. Bioink and 3D Printing

The bioink used for this experiment was gelatin methacryloyl (GelMA; Biotechnologies Ltd., Ottawa, ON, Canada). A 4% GelMA bioink was prepared by dissolving 160 mg of the solid GelMA (80% degree of substitution) in 3.6 mL of phosphate-buffered saline (PBS; Sigma Aldrich, St. Louis, MO, USA) with constant stirring at 37 °C for 20 min until a homogenous fluid was obtained. Afterward, 10 mg of the photoinitiator lithium phenyl-2,4,6-trimethybenzoyl phosphinate (LAP; Sigma Aldrich, USA) was dissolved in 1 mL of PBS separately, and 0.4 mL of this solution was added to the GelMA mixture. This was stirred for another 10 min under light-protected conditions to prevent photoinitiation. Lastly, 3 mL of the bioink was used to account for losses during handling. The bioink was added to the cell pellet and mixed slowly with a positive-displacement pipette to avoid air bubbles and foaming. Cells were incorporated at a density of 10 × 10^6^ cells/mL. The mixture was then added to a UV-blocking 3 mL cartridge (Barrel 3cc amber; VIEWEG GmbH, Kranzberg, Germany) and cooled in a 15 °C water bath for 15 min.

Meanwhile, the Celllink INKREDIBLE+ bioprinter (Celllink, Gothenburg, Sweden) was prepared and cooled down to a temperature of 16–17 °C. For the printing process, UV-blocking dispensing tips with a 22 gauge and an inner diameter of 0.41 mm were used (Dispensing tip tapered UV-blocking, black; VIEWEG GmbH, Kranzberg, Germany), with a printing velocity of 400 mm/min. The printing pressure ranged between 20–25 kPa. The pressure was affected by the slightly fluctuating temperature of the bioprinter and the exchange of cartridges.

The 3D-printed constructs were printed on a scaffold in the shape of grid into a Petri dish and were immediately crosslinked with 405 nm light for 2 min, starting during the process to ensure shape fidelity. One scaffold consisted of three layers. Four scaffolds could be printed in one Petri dish.

The constructs were detached carefully and transferred into 6-well plates covered with the enriched ASC medium and were incubated at 37 °C and 5% CO_2_ after being irradiated.

**Figure 1:**
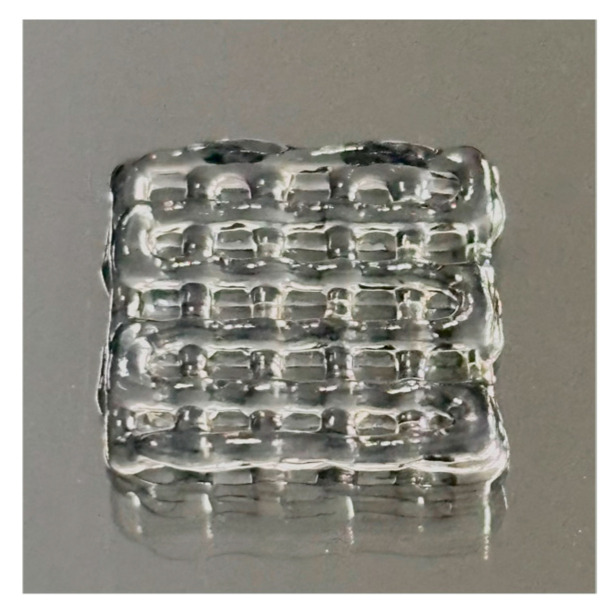
Close-up view of a 3D-bioprinted ASC-laden GelMA construct.

The constructs consisted of a 1 cm × 1 cm 3D-bioprinted scaffold comprising six parallel strands arranged with a center-to-center spacing of 2 mm (Figure 1).

### 2.3. Irradiation

Using an ISOVOLT 160 M2 X-ray tube (GE Sensing & Inspection Technologies, Ahrensburg, Germany), X-rays at a voltage of 120 kV were used to irradiate the constructs. For this study, one group of constructs was irradiated at 5 Gy and another with 2 Gy. Additionally, one group was left unexposed (0 Gy) to serve as a control group. The samples were irradiated at the focal plane of the X-ray tube in a 6-well plate, with each containing 3 scaffolds in the corresponding ASC medium. The dosage of 2 Gy was achieved at a dose rate of 120 kV and 12.2 mA for 0.5 min, and the dosage of 5 Gy was obtained at 120 kV and 21.5 mA for 0.7 min.

### 2.4. Cell Viability

#### 2.4.1. WST-8 Assay

A WST-8 assay (PromoCell GmbH, Heidelberg, Germany) was used to inspect the metabolic activity of cells in the construct over a period of 24 h, 48 h and 72 h to then compare the two irradiated groups (5 Gy, 2 Gy) with the control group (0 Gy). This demonstrated the effect of irradiation on the growth of cells. Three constructs were used for each assay. For this examination, a 24-well plate was used. Each construct was added into one well. For the assay, a reagent solution was prepared from 500 μL ASC Medium and 50 μL CCVK1 for each well. This was placed into each of the three wells. Additionally, one well was filled with just the reagent solution as a blank. The plate was then light-protected and incubated for 2 h at 37 °C and 5% CO_2_. Afterward, 100 μL of the solution was pipetted into a 96-well plate in rows of three wells. The absorbance was then measured in triplicate at 450 nm and 600 nm using the Multiscan Go and SkanIt RE for Multiscan Go 6.1 software (Thermo Fisher Scientific, Waltham, MA, USA).

#### 2.4.2. Live/Dead Assay and Fluorescence Microscopy

By using the live/dead assay, it was possible to demonstrate the survival of cells in the 3D-printed constructs after being irradiated. Two to three constructs were used for this assay. First, the constructs were washed with Hanks balanced salt solution (HBSS; Gibco Life Technologies, Thermo Fisher Scientific, Waltham, MA, USA) in a 24-well plate for 15 min in the incubator at 37 °C and 5% CO_2_. During this period, the staining solution was prepared: 8 μL calcein AM (Invitrogen, Carlsbad, CA, USA) mixed with 4 mL ADSC Medium in a 1:500 dilution, with a final concentration of 2 μg/mL, and 4 μL propidium iodide (Sigma-Aldrich) with 4 mL Medium in a 1:1000 dilution, leading to a final concentration of 1 μg/mL. The staining solution had to be protected from light. After washing the cells, 0.5 mL of calcein AM solution was added to the constructs, and the plate was incubated under a light-protecting cover for 30 min. Subsequently, the solution was removed and 0.5 mL of propidium iodide solution was added. This was left to incubate for 5 min at room temperature in the dark. After being washed again with HBSS for 15 min, the constructs were analyzed under an Olympus fluorescence microscope (Olympus IX-83, cellSens software V1.16; Olympus Corporation, Tokyo, Japan). Using an x10 objective lens (Evident Scientific, Waltham, MA, USA), six images were taken of each construct in a diagonal line across the grid. Three images were taken at each point, starting from the surface and ascending by 100 μm per image. The calcein AM colored the live cells green while the propidium iodide colored the dead nuclei red. Lastly, the fluorescence intensity was analyzed using ImageJ software version 1.54p (NIH, Bethesda, MD, USA). The measured intensity of each image was divided by the corresponding exposure time, and the mean value was calculated for each condition.

#### 2.4.3. qPCR

Quantitative Polymerase Chain Reaction (qPCR) was used to measure the gene expression fold change of the irradiated adipose-derived stem cells in our biofabricated constructs. To isolate RNA from the constructs, TRIzol^®^ (Thermo Fisher Scientific, USA) was used. Three constructs were added into a 2 mL Eppendorf Safe-Lock Tube (Eppendorf AG, Hamburg, Germany) with 1 mL TRIzol^®^. Then, the constructs were broken up slowly using a positive-displacement pipette. The mixture was then centrifuged at 12,000× *g* for 10 min at 4 °C, and the supernatant was pipetted into a new tube. After resting the tube for 5 min at room temperature, 0.2 mL of chloroform (Sigma Aldrich, Germany) was added to the sample and shaken thoroughly for 15 s. Again, the mixture was incubated for 3 min at room temperature and then centrifuged at 12,000× *g* for 15 min at 4 °C. The liquid supernatant was transferred into a new tube, and 0.5 mL of 100% isopropanol (Th. Geyer GmbH & Co. KG, Renningen, Germany) was added. Once more, the sample was kept at room temperature for 10 min and again centrifuged at 12,000× *g* for 10 min at 4 °C. After centrifugation, the supernatant was removed, and only the pellet was washed with 1 mL of 75% EtOH (Sigma Aldrich, Germany). Vortexing of the solution ensured thorough mixing before centrifuging the sample for a final time for 5 min at 7500× *g* at 4 °C. The supernatant was discarded and the pellet dried for 5–10 min at room temperature.

After this process, the RNA was cleaned with the RNeasy^®^ Mini Kit (Qiagen Gmbh, Hilden, Germany), using the manufacturer’s protocol. The NanoDrop instrument (Thermo Fisher Scientific) was then used to measure the RNA concentration and purity.

**Figure 2:**
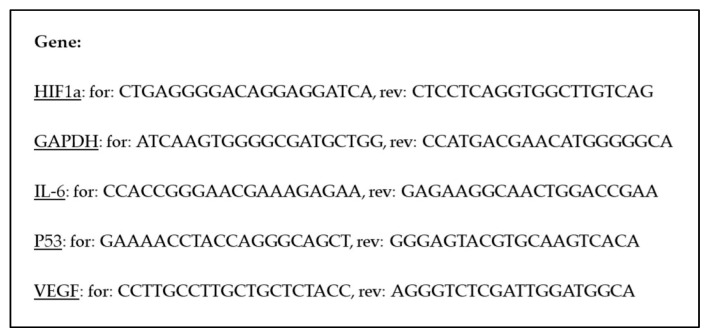
qPCR primer sequences. 5′-3′ primer sequences are shown. Genes are underlined. Abbreviations: for = forward primer, rev = reverse primer, p53 = tumor protein 53, IL-6 = Interleukin-6, HIF1a = Hypoxia-inducible factor 1-alpha. VEGF = Vascular Endothelial Growth Factor.

RNA (1 μg) was transcribed into cDNA with the QuantiTect^®^ Reverse Transcription Kit (Qiagen GmbH, Germany) following the standard protocol. For the qPCR, SsoAvancedTM Universal SYBR^®^ Green Supermix (Bio-Rad Laboratories Incorporation, Hercules, CA, USA) was used. qPCR was then performed with the CFX96 Touch Real-Time PCR Detecting System (Bio-Rad Laboratories GmbH, Germany) using primers targeting tumor protein p53 (p53), Interleukin-6 (IL-6), Vascular Endothelial Growth Factor (VEGF) and Hypoxia-inducible factor 1-alpha (HIF1a). Gyceraldehyde-3-phosphate dehydrogenase (GAPDH) was used as a reference gene (Figure 2). All the primers were purchased from the Sigma-Aldrich Corporation and were used in a final concentration of 300 nM. Relative changes were calculated using the 2^−ΔΔCq^ method with the 24 h 0 Gy sample as the reference.

The GeneRuler 100bp Plus DNA Ladder (Thermo Fisher Scientific) was used as a molecular size marker for agarose gel electrophoresis (Figure S1).

### 2.5. Immunohistochemistry

For the immunohistochemical analysis, we used the central region of the middle strand of each 3D-printed construct to ensure comparable diffusion conditions. From each construct, four serial cryosections were prepared. In total, three constructs per condition were analyzed. Sections were stained for HIF1a to evaluate hypoxia-related signaling and for Ki-67 as a marker of proliferation.

#### 2.5.1. HIF1a Staining

Staining with the HIF1a (C-Term) Polyclonal Antibody (Cayman Chemical Company, Ann Arbor, MI, USA) was performed after preparing the slides by deparaffinization. After pressure cooking the slides in citrate buffer solution (S1699; Aligent Dako, Santa Clara, CA, USA) at 121 °C for 7 min, the slides were washed three times with Tris Buffer and marked with a PAP Pen (Zytomed Systems, Bargteheide, Germany). Afterward, the slides were blocked with Peroxidase-Blocking solution (Dako REAL; Aligent Dako, USA) for 15 min. Following the washing steps, a protein block (Aligent Dako, USA) was added for 7 min, followed by incubation with 10% goat serum for 30 min. After diluting the antibody with antibody dilutant (Zytomed Systems, Germany) according to the manufacturer’s instructions, it was added to the slides and incubated first for 1 h at room temperature and then overnight at 4 °C.

The next day, the slides were washed and the secondary antibody rabbit/mouse HRP (Dako REAL envision; Aligent Dako, USA) was added for 30 min. The slides were stained with 3,3’-Diaminobenzidine (DAB) (Aligent Dako, USA) under constant evaluation and immediately washed to stop the process. Counterstaining was performed using Mayer’s hematoxylin diluted 1:10. Sections were mounted with Aquatex ^®^ (Merck, Darmstadt, Germany) and dried for 1 h.

#### 2.5.2. Ki67 Staining

The Ki67 rabbit monoclonal antibody (Zytomed Systems, Germany) staining was conducted after deparaffinization of the slides. The slides were incubated in a 95 °C water bath in citrate buffer solution (S1699; Aligent Dako, USA) for 30 min. Following this antigen retrieval step, the primary antibody was mixed according to the manufacturer’s instructions and applied to the slides. The slides were immediately placed at 4 °C for overnight incubation. All subsequent steps followed the procedures described above.

The slides were evaluated under the microscope (Keyence BZ-X810, Keyence, Neu-Isenburg, Germany).

### 2.6. Material Characterization of Hydrogels

#### 2.6.1. Rheology

Rheological measurements were performed using a DHR-3 rheometer (TA instrument, Newcastle, NC, USA) equipped with a 20 mm cone plate. The measurement gap was set to 56 μm according to the manufacturer’s specifications. A solvent trap was used to prevent sample drying and unintended UV-induced crosslinking.

Samples were stored at 37 °C between measurements and pipetted onto the pretempered rheometer. Prior to each measurement, samples were cooled from 37 °C to 15 °C at a rate of 2 °C/min and equilibrated at 15 °C for 4 min, resulting in a total cooling protocol of 15 min. All rheological measurements were subsequently carried out at a constant temperature of 15 °C.

For each sample, the following measurement protocol was applied in sequence: temperature treatment, frequency sweep, amplitude sweep and a second frequency sweep. The initial frequency sweep was conducted within the linear viscoelastic region at a constant deformation of 1%, which was validated by the amplitude sweep (0.01–100% deformation). The frequency sweeps were used to determine the storage modulus (G′), loss modulus (G″) and complex viscosity as a function of angular frequency (rad/s).

To assess the structural recovery after large deformations, a second frequency sweep was performed immediately after the amplitude sweep using identical measurement parameters. Comparable rheological responses between the first and second frequency sweeps were interpreted as evidence of good material recovery.

For data presentation, results from repeated measurements were averaged, and standard deviations were calculated and displayed as error bars.

#### 2.6.2. Cryo-Scanning Electron Microscopy

The samples were rapidly frozen in slushed nitrogen at −210 °C after placing them between aluminium plates (d = 3 mm) with a 2 mm notch for sample fixation. All the following transfer steps were performed at −140 °C with an EM VCT100 cryo-shuttle (Leica Microsystems, Wetzlar Germany). To generate a freshly fractured hydrogel surface, one of the aluminium plates was knocked off and freeze-etched for 15 min at −85 °C under high vacuum (<1 × 103 mbar) in a Sputter Coater machine (ACE 400; Leica Microsystems). Afterward, samples were sputtered with 3 nm platinum and transferred to the SEM chamber (Crossbeam 340; Zeiss, Oberkochen Germany). Images of the hydrogel surface morphology were taken at −160 °C using an acceleration voltage of 8 kV.

### 2.7. Supplementary Experiments

To compliment the 3D experiments, a separate experimental run on 2D cultures was performed. A total of 50,000 cells were seeded into three wells of a 24-well plate. Cells were subjected to the same radiation dose and analysis as used for the 3D constructs.

## 3. Statistical Analysis

All experiments were performed in triplicate and were analyzed using Graph Pad Prism 10.2.0 software (GraphPad Software Inc., Solana Beach, CA, USA). To test for normally distributed data, we used the Shapiro–Wilk test. Differences between groups were analyzed using an analysis of variance (ANOVA), followed by Tukey post hoc tests to identify pairwise differences between the groups at different timepoints. For non-normally distributed data, the Kruskal–Wallis test was performed, followed by Dunn’s post hoc test.

The graphical abstract was created using BioRender R.S. (2025). https://BioRender.com/wzdlbhg.

## 4. Results

In this study, we performed WST-8 assays, live/dead staining and qPCR to evaluate the cell viability, cellular response to irradiation and gene expression under different experimental conditions.

### 4.1. Ink Characterization

**Figure 3:**
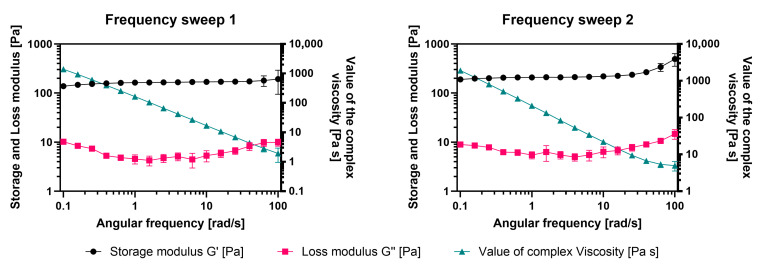
Rheological properties of the GelMA bioink.

The frequency-sweep measurements revealed a predominantly solid-like behavior of the material across the investigated angular frequency range. In all samples, the storage modulus (G′) consistently exceeded the loss modulus (G″), indicating a gel-like structure (Figure 3). Both moduli showed a frequency-dependent increase, consistent with ongoing gelation at 15 °C during the measurement timeframe. The complex viscosity decreased with increasing angular frequency, displaying typical shear-thinning behavior. Amplitude-sweep measurements covering deformations from 0.01% to 100% confirmed the linear viscoelastic region at 1% deformation, which was subsequently used for the frequency-sweep experiments. Despite exposure to large deformations during the amplitude sweep, the material maintained its structural integrity.

The second frequency sweep performed after the amplitude sweep closely overlapped with the initial frequency sweep. No measurable differences were observed in G′, G″ or complex viscosity between the two measurements. This reproducibility indicates effective structural recovery of the gel following large deformations. Overall, the rheological data demonstrate a stable, elastic-dominated gel with time-dependent stiffening at 15 °C and good recovery behavior after mechanical stress.

### 4.2. Cryo-Scanning Electron Microscopy

**Figure 4:**
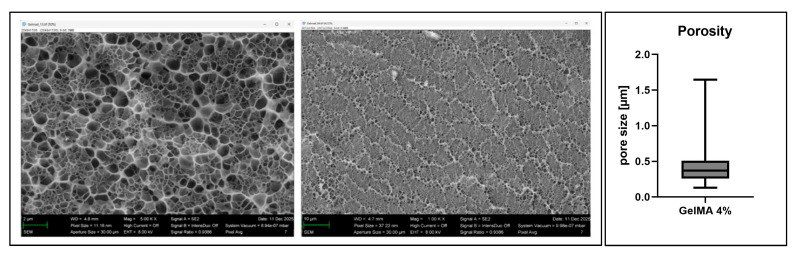
Porosity of 3D scaffolds made from 4% GelMA.

The 4% GelMA constructs exhibited a mean pore size of 0.4281 μm with a standard deviation of ±0.2352 μm (Figure 4).

### 4.3. WST-8 Assay

**Figure 5:**
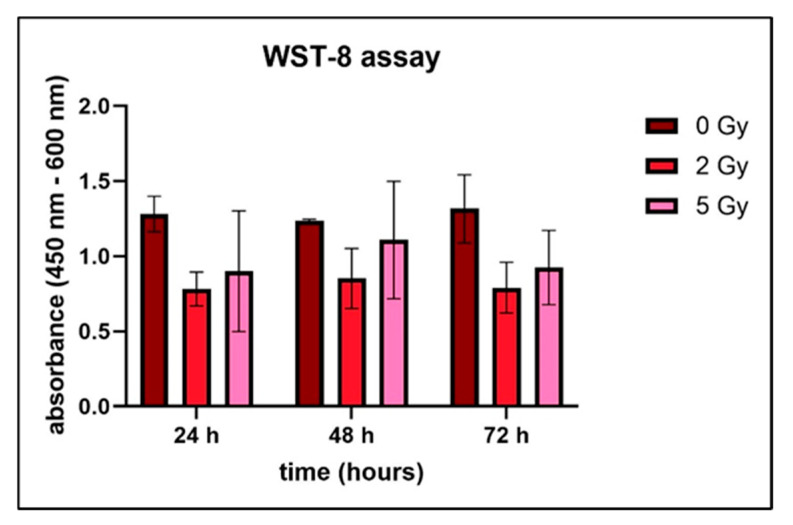
Combined metabolic activity of adipose-derived stem cells within bioprinted constructs irradiated with 5 Gy, 2 Gy and 0 Gy. Constructs were measured at 24 h, 48 h and 72 h (*n* = 3). Data are presented as the mean ± standard deviation. No significant differences were detected. Additional experiments were conducted using 2D cultures. Within the single 2D experiment, proliferation was not affected by the different radiation dosages or significantly different when compared with the control group.

The metabolic activity and growth of the cells in the construct was measured after 24 h, 48 h and 72 h (Figure 5). The results of the Kruskal–Wallis test revealed that there were no significant differences when the irradiated groups were compared with the control group. This indicates that cell viability and metabolic activity were not affected by the radiation doses that were used, showing that the cells survived and maintained a normal metabolic function even after exposure to radiation.

### 4.4. Live/Dead Assay

**Figure 6:**
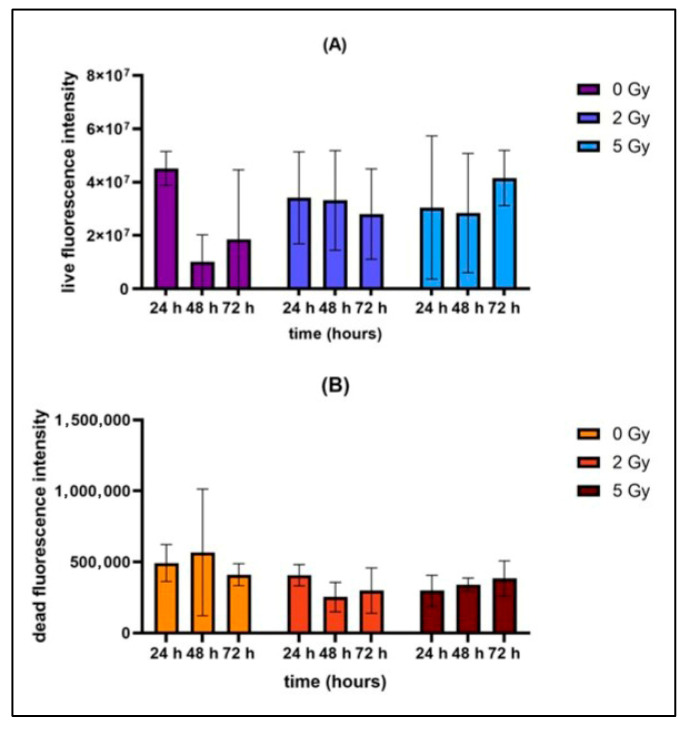
Cell viability of biofabricated constructs irradiated with 0 Gy, 2 Gy and 5 Gy (*n* = 3). The fluorescence intensity was measured at a time period of 24 h, 48 h as well as 72 h. Data are presented as the mean ± standard deviation. (**A**) Fluorescence intensity of live cells. (**B**) Fluorescence intensity of dead cells. No significant differences were detected.

**Figure 7:**
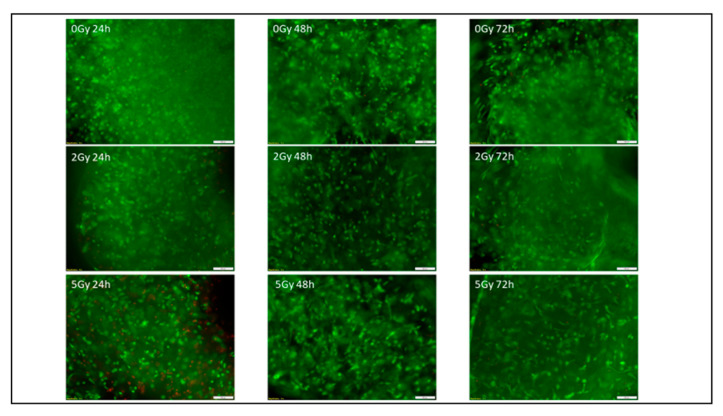
Cell viability of 0 Gy, 2 Gy and 5 Gy irradiated adipose-derived stem cells in GelMA printed constructs, analyzed under an Olympus fluorescence microscope after 24 h, 48 h and 72 h (*n* = 3). Green indicates live cells, red indicates dead cells. Scale bar = 100 µm. The results of the live/dead assay revealed no detectable effects of irradiation on cell viability under the conditions tested. Since only one experimental run was carried out, statistical analysis was not possible.

To determine the survival of the ASCs in the biofabricated samples, a live/dead assay was performed after 24 h, 48 h and 72 h. Due to the normally distributed data, analysis of variance (ANOVA) followed by the Tukey post hoc test were used to analyze the results. When the two irradiated groups were compared with the control group during the 72 h time period, no significant differences were found (Figure 6 and Figure 7). The radiation dosages of 5 Gy and 2 Gy did not induce increased or decreased cell death or impair cell proliferation compared with the control group during the measured time period.

### 4.5. qPCR

**Figure 8:**
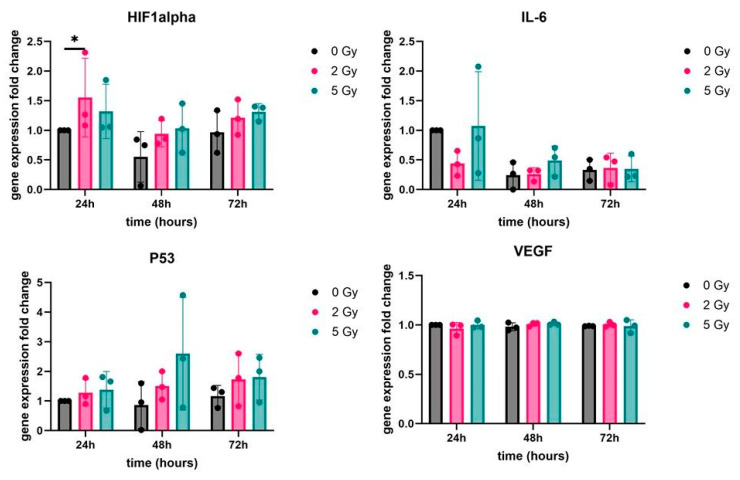
Gene expression fold change in HIF1a, IL-6, p53, and VEGF from the adipose-derived stem cells irradiated with 0 Gy, 2 Gy and 5 Gy in the biofabricated constructs. The gene expression was tested after 24 h, 48 h, and 72 h (*n* = 3). Data are represented as the mean ± standard deviation. * *p* ≤ 0.05.

qPCR was performed to quantify the gene expression levels of p53, IL-6, VEGF and HIF1 alpha relative to the housekeeper gene GAPDH. For this experiment, the constructs were irradiated and collected after 24 h, 48 h and 72 h to be analyzed at once. The results were analyzed by Dunn’s post hoc analysis, which detected a significant difference (*p* = 0.0458) in the HIF1a gene expression fold change between the 0 Gy control group and the irradiated 2 Gy group after 24 h (Figure 8). The expression in the 2 Gy group was 1.5-fold higher than that in the control group. There was no significant difference found after 48 h and 72 h. No significant difference was detected in the expression levels of the other three other genes (p53, VEGF and IL-6) compared with the control group over the 72 h time period.

Melt-curve analysis and agarose gel electrophoresis were carried out as supplementary tests and confirmed the specificity of all the qPCR primer sets, demonstrating single well-defined amplification products (Figure S2).

### 4.6. Immunohistochemistry

**Figure 9:**
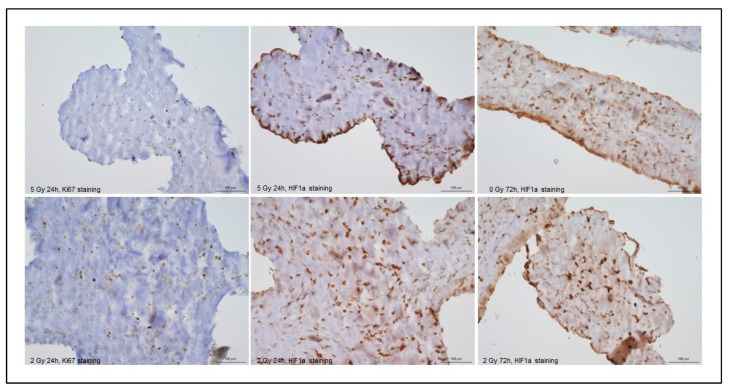
Immunohistochemical staining for Ki67 and HIF1a in the central region of the middle strand of each 3D-printed construct.

Immunohistochemical staining for Ki67 demonstrated consistently low proliferative activity of ASCs across all irradiation groups and timepoints (Figure 9). In particular, the 5 Gy constructs displayed high apparent cell density at the periphery of the construct, but Ki67-positive nuclei remained sparse throughout the construct. HIF1a staining demonstrated dose- and region-dependent patterns. At 24 h, strong nuclear HIF1a accumulation was present predominantly in the central regions of the 2 Gy constructs, with markedly weaker signal toward the periphery. At 72 h, both 0 Gy and 2 Gy constructs showed cell-rich peripheral zones; however, the Ki67 staining remained low despite the presence of viable cells.

## 5. Discussion

With more than 50% of oncologic patients receiving radiotherapy and nearly 20 million new cases of cancer every year, radiotherapy is a well-established part of clinical cancer therapy [20,21]. The high prevalence of patients and the frequent use of radiotherapy pose considerable challenges to the successful integration of grafts or flaps in reconstructive surgery. Complications like endothelial cell injury, fibrosis and inflammation are observed as typical long-term side effects, even though maintaining vascular integrity is vital for flap survival. Fibrosis, also known as chronic radiation-induced fibrosis, tends to present 4 to 6 months after radiotherapy and continues to develop for years [21]. This can impair flap or graft integration by reducing vascularization and perfusion, thereby increasing the risk of ischemia and compromising tissue survival. Radiation injuries to the skin might seem to cover small areas but actually extend deep into the soft tissue, even reaching the underlying muscles and bones [21]. Also, given the widespread presence of adipose tissue in the body, its exposure and damage during radiotherapy is unavoidable. For this reason, strategies for preserving or restoring cellular proliferative potential as well as enhancing local microcirculation are currently a major focus of research. In our previous studies, the application of stem cells, including adipose-derived stem cells, demonstrated anti-inflammatory effects in irradiated tissue, suggesting potential protective effects [22]. ASCs are known to exert these effects through their paracrine characteristics. Under hypoxic conditions, they secrete various growth factors, such as angiogenic factors, that stimulate the recovery of damaged tissue. ASCs are also sensitive to growth factors and inflammatory stimuli that enhance their regenerative activity through proliferation, migration and paracrine signaling [23,24].

Until now, most research on this topic has been conducted on 2D cell culture models. To overcome this limitation, this study investigates the effects in 3D cultures, providing a more physiologically relevant system. The use of 3D culture systems enables more accurate in vitro simulation of in vivo-like cellular behavior. In the context of adipose-derived mesenchymal stem cells, transitioning from 2D to 3D culture has been associated with an up-regulation of metabolic activity, potentially reflecting increased cellular energy demands under 3D growth conditions [25]. Furthermore, ASCs cultivated in 3D may enhance their biological activity and efficacy by increasing the release of paracrine signals or by interacting with and remodeling the extracellular matrix. All these processes can facilitate cell survival, migration, proliferation, differentiation and angiogenesis [26]. Cells in 3D culture are in close association with each other and are in contact with a greater number of cells compared with those in monolayer culture. This might improve intercellular signaling, as more molecules must be present in monolayer cultures to ensure effective communication [27,28]. In this study, GelMA was used as a bioink to support cell growth and to maintain a robust structure. It was used due to its high biocompatibility and degradability [29]. Since it is derived from gelatin, it can mimic the extracellular matrix, which supports cell adhesion via RGD motifs, proliferation and differentiation. It allows for the simulation of artificial tissues. Schipka et al. demonstrated that differences in gelatin binding, composition and crosslinking of gelatin-based bioinks could influence cell migration and spreading behavior [30]. Additionally, GelMA provides favorable conditions for photo-crosslinking, which allows the creation of layered 3D constructs [31]. Previous studies have shown that ASCs remain viable and functional within GelMA-based scaffolds. Guo et al. demonstrated that ASCs in GelMA adhered, proliferated and retained their differentiation potential in vitro, which indicates that any functional decline or recovery after irradiation likely reflects a cellular response rather than scaffold failure [32].

Our results showed that cell survival was high after 24 h, indicating that the printing process with GelMA was successful and a suitable choice, with minimal shear forces produced during printing that could damage or deform the cells. Cell exposure to irradiation causes cellular stress, DNA damage, autophagy and the generation of reactive oxygen species (ROS). Although irradiation does not directly reduce oxygen levels, ROS can cause oxidative damage and act as signaling molecules that activate pathways involved in cell survival and damage repair to alleviate the damage [33]. Among these pathways, hypoxia-inducible factors (HIFs) are key mediators of the cellular stress and hypoxia response. This activates target genes involved in adaptive mechanisms that help cells survive and adapt. These mechanisms include cell survival and proliferation, glucose metabolism and angiogenesis [34].

Studies have shown that ionizing radiation generates ROS in proportion to the radiation dose, which can act as signaling molecules influencing transcriptional responses at a mildly increased dosage [35]. Lall et al. demonstrated in fibroblasts that ROS stabilize the HIF1a protein by preventing its degradation and that low-dose irradiation can induce HIF1a expression via transcriptional and translational pathways [35,36]. While our data measure HIF1a mRNA in ASCs, similar regulatory mechanisms may contribute to its expression in our system without exposing the biofabricated constructs to hypoxia. Although fibroblasts and ASCs are distinct cell types, both are mesenchymal in origin and share phenotypic similarities, supporting the relevance of this study for examining HIF1a regulation in ASCs [37].

In the present study, we observed a selective 1.5-fold increase in HIF1a mRNA expression 24 h after irradiation with 2 Gy, while the expression of the other genes analyzed, p53 and IL-6, were not changed significantly when compared with the control group. This could suggest that radiation-induced ROS cause oxidative stress, which stabilize HIF1α and lead to increased transcriptional activity, consistent with previously described hypoxia-like responses under normoxic conditions.

Another reason for the lower irradiation sensitivity of the 3D-printed cells might be local hypoxia. Hypoxia can enhance the radioresistance of cells [38,39] and has been observed in dense 3D-cultured multicellular spheroids [40]. Hypoxia in 3D cultures is common at their center due to increased diffusion distances [41]. Therefore, larger 3D constructs show steep oxygen gradients, which produce hypoxic or anoxic cores that alter cellular metabolism and gene expression [41]. This could exacerbate HIF1a mRNA expression via the pre-existing oxygen gradient, which produces local hypoxia-like microenvironments. The pore size of our constructs indicate a dense network structure that permits diffusion, potentially contributing to spatial gradients within the construct. Increased HIF1a expression at 2 Gy at 24 h was detected both at the mRNA level and by the enhanced HIF1a staining in the construct core, suggesting that moderate irradiation amplifies the hypoxic response of ASCs within the 3D construct. The pronounced central HIF1a signal together with the reduced staining at the periphery could indicate that irradiation enhances the cellular hypoxia response in this zone. As Antoni et al. describe, 3D cultures develop oxygen, nutrient and waste gradients that are absent in 2D systems [42]. These gradients generate microenvironmental differences within the construct. This concept is demonstrated in our histological findings, where ASCs consistently accumulated at the construct edge. This was consistent after 72 h in both the 0 Gy and the 2 Gy groups. This concept was most pronounced in constructs that were irradiated with 5 Gy. The outer zone offers more favorable nutrient availability, enabling better survival. Although Ki67 staining showed low proliferative activity across all conditions, the WST-8 and live/dead assays confirmed robust metabolic activity and high cell viability throughout all radiation dosages, even at 5 Gy. This may reflect an adaptive response of ASCs to combined radiation-induced stress and oxygen diffusion gradients in the 3D culture, while the cells remain largely non-proliferative but metabolically active. Rusin et al. demonstrated that ionizing radiation activates DNA damage checkpoints, which enforces G1/S and G2/M arrest to allow DNA repair [8]. This checkpoint activation suppresses proliferation. Our findings that ASCs survive 5 Gy but show minimal Ki67 staining align with this concept.

Studies have demonstrated that hypoxia improves the function of adipose-derived stem cells, including their survival, proliferation, migration and differentiation, since it apparently creates a more realistic environment for the ASCs [43,44,45,46]. Most importantly, it increases their paracrine activity and can up-regulate VEGF expression under hypoxia, promoting angiogenesis [25], which is critical for the survival and successful integration of grafts or flaps in reconstructive surgery.

In contrast, a higher irradiation dosage of 5 Gy did not further increase the gene expression of HIF1a in comparison with the control group. The results suggest that higher radiation dosages do not strongly affect HIF1aexpression, suggesting a non-linear response. It is also possible that 5 Gy induced a stronger initial HIF1a expression, which was then reduced more quickly due to a more pronounced cellular response. A higher radiation dosage did not overwhelm the cells but, instead, activated protective mechanisms, implying that HIf1a has an adaptive, not cytotoxic, effect and proves that the pre-existing oxygen gradient in the 3D culture enhances the radioresistance of the cells.

p53 is a tumor suppressor gene that acts as a regulator of DNA-damage responses [24]. As mentioned above, there was no significant difference in its gene expression compared with the control group, which could suggest that the ASCs tolerated the radiation doses well. Even when irradiated with an increased dose of 5 Gy, it did not cause apoptosis, indicating that protective mechanisms might have prevented this. It could also indicate that early or transient activation may have been missed due to the sampling times being at 24 h, 48 h and 72 h.

The pro-inflammatory cytokine Interleukin-6 (IL-6) plays a central role in inflammatory responses and is induced by various inflammatory stimuli. It is also transiently expressed in response to environmental stress factors such as tissue injury [47]. However, in our experiments, it showed no significant change within the 3D constructs nor in the 2 Gy or 5 Gy groups compared with the control group, indicating either that radiation did not strongly trigger an inflammatory response in the used construct at the tested time or that a transient activation may have been missed, as suggested earlier.

Although ASCs are generally described as expressing VEGF at high levels, VEGF mRNA expression did not increase after irradiation, suggesting that irradiation and the 3D environment did not further induce it. This is also consistent with previous findings by Fekete et al., who reported that MSCs do not necessarily increase VEGF expression following irradiation, despite activation of other stress pathways [48].

One study demonstrated that in irradiated adipose-derived stem cells, the up-regulation of the majority of genes responsible for DNA damage and repair, apoptosis and cell cycle regulation disappeared within 6 h after irradiation, indicating that most of the DNA-damage response occurs within the first 6 h after irradiation [49]. This pattern is consistent with our prior reports showing that radiation-induced transcriptional responses of DNA-damage repair, apoptosis and stress-related genes in ASCs are typically transient, peaking within the first few hours and returning to the baseline ~6 h after irradiation. Thus, the absence of p53 and IL-6 changes at 24 h likely reflects the resolution of an early DNA-damage response.

The results of the live/dead assay and WST-8 assay showed no significant apoptosis or proliferation changes in the irradiated groups compared with the control group during the statistical analyses. The microscopic fluorescence pictures of the live/dead assay (Figure 3) show no increase in dead cells even after irradiation with 5 Gy. This suggests that the radiation dosages that were used in this study were well tolerated. The cell population recovered or resisted radiation stress over the 72 h time period instead of causing cell death.

As mentioned above, irradiation with 2 Gy has been reported in multiple studies to reduce the proliferation rate of ASCs cultured in 2D [8,9,10]. However, in our experiments in 3D cultures, both the live/dead staining and WST-8 assays showed no significant differences in cell viability or metabolic activity after either the 2 Gy or 5 Gy irradiation during the 72 h time period. This may reflect differences in culture architecture, as 3D cultures create oxygen and nutrient gradients that can alter stress responses and enhance cellular resilience. Because previous studies report divergent effects of irradiation on ASC proliferation in 2D cultures, we performed one experimental run of supplementary 2D culture experiments to clarify how our cells respond under standard culture conditions. These experiments were conducted only once and therefore cannot be considered conclusive. Consistent with a general tendency [50,51], our 2D-cultured ASCs remained viable across all radiation doses. This finding highlights the radioresistance of this cell group. The outcome is also consistent with the observation of Galarza et al., who demonstrated that cellular behaviors measured in 2D cannot be directly compared with 3D environments due to fundamental differences in their microenvironment, motility and stress responses [52]. In our previous studies we demonstrated that 3D-cultured keratinocytes were negatively influenced by irradiation, supporting the idea that the results were not related to the experimental setup [53].

Moderate irradiation at these doses may only induce transient stress that does not impact survival or metabolic activity in a short period of time, consistent with the results of the unchanged P53 and IL-6 expression observed in our study. These results suggest that adipose-derived stem cells in 3D cultures can tolerate moderate irradiation without acute effects on viability. Evidence from a study indicates that even after irradiation with doses up to 8 Gy, the irradiated ASCs maintained their differentiation potential, showing the functional and phenotypical stability of ASCs after exposure to irradiation [49].

This observation is further supported by another study showing that ASCs cultured in 3D environments demonstrate enhanced differentiation potential and exhibit greater resistance to ionizing radiation compared with their 2D-cultured monolayer counterparts [39].

## 6. Conclusions

In conclusion, our results indicate that 3D-cultured adipose-derived stem cells maintain high viability after moderate irradiation, suggesting radioresistance. These findings highlight the importance of 3D cultivation as a more physiological model. Further research is needed to investigate how spatial heterogeneity within the 3D culture influences ASCs’ behavior. ROS dynamics should also be further explored to fully understand the impact of irradiation on 3D-cultured ASCs.

## Data Availability

The original contributions presented in this study are included in the article/Supplementary Materials. Further inquiries can be directed to the corresponding author.

## Supplementary Materials

The following supporting information can be downloaded at: https://www.mdpi.com/article/10.3390/bioengineering13010025/s1, Figure S1. Agarose gel electrophoresis results. Figure S2. qPCR melt curves of HIF1a, IL-6, p53 and VEGF.
