# Supplementary material for: Engineering Resilience: How Irradiation Strategies Influence 3D-Bioprinted Adipose Stem Cells
Source: Bioengineering (Basel). 2025 Dec 26;13(1):25. doi: 10.3390/bioengineering13010025 (PMC12837484; doi:10.3390/bioengineering13010025)
Supplement: Supplementary file 1 [file bioengineering-13-00025-s001.zip › bioengineering-4007379-supplementary.pdf]

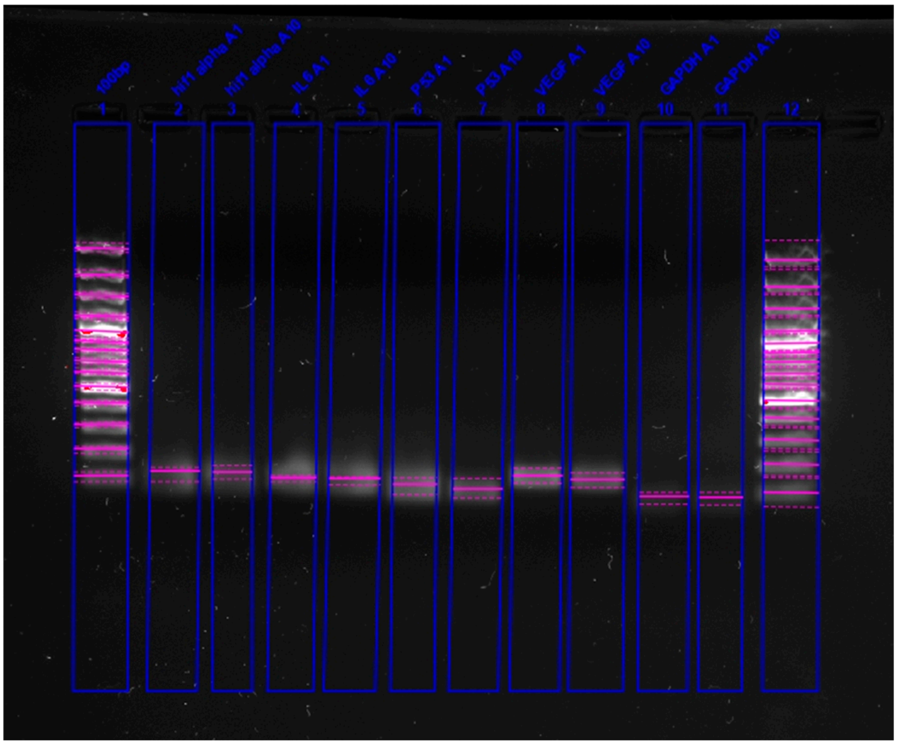

Supplementary Figure S1. Agarose gel electrophoresis results

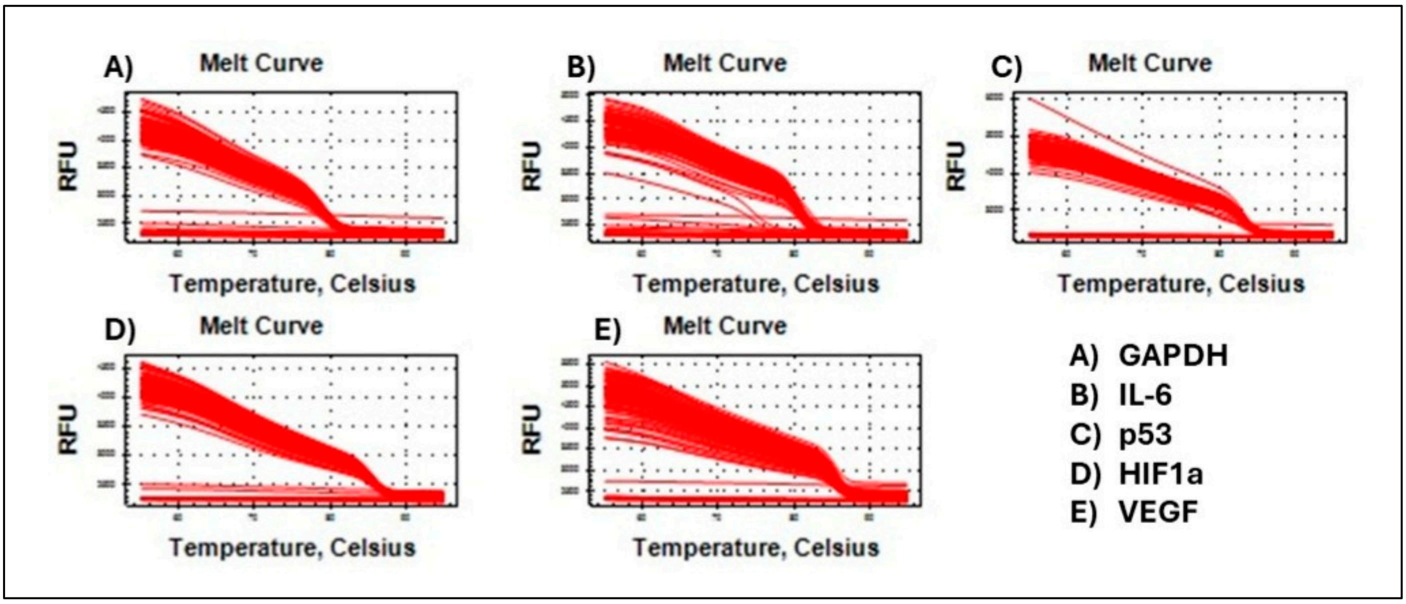

Supplementary Figure S2. qPCR melt curves of HIF1a, IL-6, p53 and VEGF.
